# 

**Published:** 1870-12

**Authors:** 


### CONTENTS.

ORIGINAL COMMUNICATIONS.

Report on Operative Dentistry .... 505
Hemorrhage After Extracting .... 516
Report on Dental Pathology and Surgery .... 517
Discussions on Dental Pathology and Surgery .... 526
Fees .... 530
Artificial Teeth .... 532
Office Notes .... 535
Discipline of Head, Heart, and Hand .... 538

SELECTIONS.

Notes on Chloral .... 541
Early History of Surgeons .... 542
A New Method of Effectually Remedying the Defect of Hare-lip .... 543

EDITORIAL.

Anæsthesia in Dental Practice .... 545
Ohio State Dental Society .... 547

